# Sunscreen Safety and Efficacy for the Prevention of Cutaneous Neoplasm

**DOI:** 10.7759/cureus.56369

**Published:** 2024-03-18

**Authors:** Jonathan R Raymond-Lezman, Suzanne I Riskin

**Affiliations:** 1 Department of Foundational Sciences, Nova Southeastern University Dr. Kiran C Patel College of Osteopathic Medicine, Clearwater, USA

**Keywords:** ingredients, skin cancer, melanoma, general dermatology, erythema, uva protection factor, uva protection, sunscreen, spf

## Abstract

Sunscreen is widely used for ultraviolet radiation protection. Studies validate sun protection factors (SPFs) to prevent ultraviolet (UV)-induced skin changes such as melanoma and squamous cell carcinoma (SCC). Few studies examine sunscreen’s impact on endocrine and immune system dysregulation, the production of radical oxygen species, and whether the ingredients deteriorate under prolonged exposure. We present an investigation of sunscreen labels and how ingredients impact sun safety and human health. A review of the literature was conducted using Embase and PubMed to examine sunscreen safety, efficacy, and use to prevent UV-induced skin damage. Increasing sunscreen reapplication, wearing protective clothing, and limiting exposure can reduce the incidence of skin cancer. Inorganic sunscreens form barriers to block UV light, but without titanium dioxide (TiO_2_), they may not be advantageous due to their low UVA protection. Organic sunscreens absorb into the skin and provide a better feeling after application. Octocrylene and avobenzone are stable and provide UVA and UVB protection with minimal adverse effects. Oxybenzone is harmful to the neuroendocrine system and should be avoided. Titanium dioxide works for broad-spectrum UV protection and offers minimal adverse effects. Octocrylene and avobenzone are organic sunscreen ingredients that also provide a better feeling on the skin after application, which enables higher rates of use. Oxybenzone should be avoided.

## Introduction and background

One of the earliest sunscreens was developed in 1935 by Eugene Schueler, the founder of L’Oreal. He used benzyl salicylate to create a tanning oil with sun protection measures. Ambre Solaire, now a Garnier product, even marketed Scheuler’s original formula as being able to help “tanning five times faster without burning” [1]. Today, there are hundreds of types of sunscreens sold with varying amounts and types of ingredients used, all with assorted degrees of efficacy.

Sun protection factor (SPF) is the amount of ultraviolet (UV) radiation the sunscreen can absorb or reflect. The SPF number refers to an inverse relationship regarding the amount of UV radiation the person applying the product will be exposed to during and immediately after sunscreen application. For example, using SPF 50 sunscreen protection on the skin will effectively work against UVB radiation for 50 times the amount of radiation exposure compared to unprotected skin [2]. The higher the SPF, the longer the duration of protection. 

Sunscreens are available in a variety of ingredients, and the varying chemical compositions provide different mechanisms of action for protection. However, they each have different adverse effects, which should weigh against their protective benefits. Determining which sunscreen is right for a particular person does not contain a “one size fits all” criterion.

## Review

This review's goals were to summarize relevant published research regarding sunscreen, skin cancer, and other UV-induced skin conditions and their relation to one another. It mainly focuses on sunscreen ingredients, their indications and contraindications, adverse effects, and safety, while highlighting areas that need further research.

Search strategy

**Figure 1:**
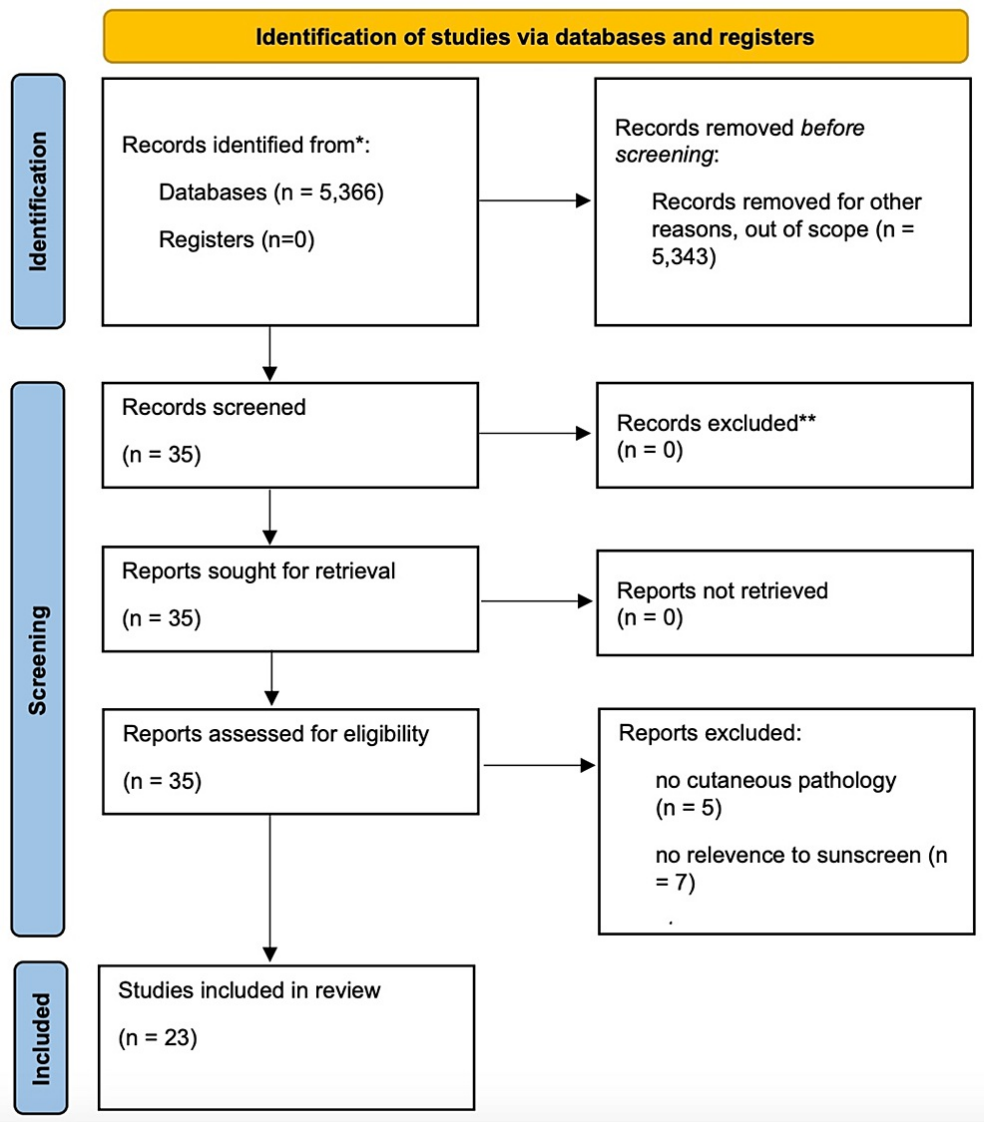
Preferred reporting items for systematic reviews and meta-analysis (PRIMSA) search strategy

Peer-reviewed articles were searched in two databases: PubMed and Embase. The terms “UVA protection factor,” “melanoma AND sun protection factor,” “sun protection factor AND ingredient,” “UV exposure skin cancer,” “sunscreen ingredients,” “octinoxate toxicity,” “sun protection factor,” and “UVR light.” These search criteria generated 5,366 peer-reviewed articles. A total of 23 articles were used in this review. Figure 1 shows the preferred reporting items for systematic reviews and meta-analysis (PRIMSA) for the search strategy.

Pathology of Cutaneous Neoplasm and UV Radiation-Induced Damage

Skin cancer is the most diagnosed cancer in the United States (US), with melanoma being the fifth most common cancer diagnosis overall [3,4]. The American Cancer Society estimates that 45% of melanoma deaths can be prevented with proper UV precautions. However, in a 2018 panel with 3,000 Americans, only 38.8% confirmed using sunscreen on their face, neck, and chest while outside in the sun. Only 19.9% applied it to their entire sun-exposed body [3].

There are three main groups of ultraviolet radiation: UVA (400-315 nm), UVB (315-280 nm), and UVC (280-100 nm). Only UVA and UVB can penetrate the ozone layer. UVB light exposure is highest between 10 a.m. and 3 p.m. and is pathologically marked by the production of sunburn (erythema). UVA exposure is highest between 8 a.m. and 5 p.m. and does not produce erythema [5]. While UVB light is 1,000 times more potent and toxic than UVA light, UVA light exposure is 20-40 times higher [4].

UVA exposure increases the risk of melanoma, reactive oxygen species (ROS), carcinogenesis, deoxyribonucleic acid (DNA) damage, and photoaging [3,5]. UVA-induced DNA damage occurs from thymine and pyrimidine-pyrimidone dimers and targets p53, which normally enables cell survival [6]. UVA also induces oxidative damage to cells and is repaired through base excision at lower rates with higher melanocyte mutations [4]. UV-induced photoaging occurs from repeated sunburn with a response of wound-healing behavior from the body. That aging process results in the onset of events that promote extracellular matrix (ECM) remodeling, such as matrix metalloproteases (MMP), collagen cross-linking, and elastin degradation. These events promote tissue remodeling, wrinkling, and “visible aging.” Langerhans cells are depleted upon exposure to UV radiation. Immature dendritic cells and monocytes replace them, leading to immunosuppression [6]. Immunosuppression is a major factor in the development of carcinomas, even without preceding erythematous changes in the skin from sun exposure, due to PTCH, SMOH, and TP53 mutations [3,6].

Melanoma usually arises de novo due to exposure to UV radiation, including sun exposure and solariums, which can eventually cause episodes of sunburn. Squamous cell carcinoma (SCC) and basal cell carcinoma (BCC) have been linked to chronic UV exposure, regardless of sunburn [7,8].

Sunscreen Ingredients, Adverse Effects, and Safety

Sunscreens today are generally made of two categories of ingredients: organic and inorganic. Inorganic sunscreen ingredients include zinc oxide (ZnO) and titanium dioxide (TiO_2_). Their mechanism of action is simple: they form a physical barrier on the skin and block UV radiation from penetrating into the skin [5]. Inorganic filters tend to leave a white residue or haze on the skin, which can cause a reflexive decrease in the amount of product applied. In recent years, the technology to create nanoparticles of inorganic sunscreens has revolutionized their appearance and feel on the skin. They now produce a little white residue, or haze [5].

One danger of TiO_2_ nanoparticles is through inhalation when applying aerosolized sunscreen. Inhaled particles mainly stay in the upper airways. The particles that do make it into the lung tissue are expelled through the mucociliary escalator and Kupffer cells. Due to the low clearance rate, it is estimated that 10% of the particles remain within the lungs. These particles can also migrate through the lung tissue into other systemic organs. The effects include irritation, inflammation, and impaired vasodilation of the systemic microcirculation due to endothelial dysfunction mediated by the ROS generated from TiO_2_ particles. Other products containing TiO_2_, such as lip balm, show minimal absorption through oral ingestion. Particles were found in the gastrointestinal tract, but only at 0.05% concentration after one week [9].

Organic sunscreen ingredients are compounds manufactured to absorb into the skin completely. The most frequently used are homosalate, octocrylene, oxybenzone, avobenzone, and octinoxate. Organic filters capture UV radiation and transform it into energetic radiation greater than the incident radiation, either as visible light or infrared radiation. Organic filters are generally more popular to wear due to their feel on the skin as compared to inorganic filters [5]. Organic sunscreens have a drawback in comparison to inorganic sunscreens - potential UV-induced degradation. An ideal sunscreen should be able to convert from excited to ground state as rapidly as possible to be able to absorb more radiation again [10].

Avobenzone demonstrated no long-lived radiative decay. The avobenzone molecules achieve ground state from the excited state just as quickly as they go to the excited state, thus dissipating UV radiation into vibrational energy (heat). A problem that occurs with avobenzone, compared to octocrylene, is that it does not return entirely to the ground state [10]. When avobenzone and octocrylene are combined, octocrylene stabilizes avobenzone from reaching the reactive enol triplet state [10,11]. Avobenzone degraded quickly on its own when exposed to UVA radiation and had deleterious effects on the other sunscreen ingredients [10].

The drawbacks of organic sunscreens vary. Many studies have shown that some organic sunscreen compounds disrupt the endocrine system. Oxybenzone (benzophenone-3) displays osteogenic, anti-osteogenic, and anti-androgenic effects in in vitro studies [12]. Experimental studies show that oxybenzone and enzacamene (3-(4methylbenzylidene)camphor) are widely absorbed through the skin into the systemic circulation. These highly lipophilic compounds have high bioaccumulation within the body. Oxybenzone levels have been measured in breast milk, urine, and adipose tissue. They can cross the placenta, leading to increased birth weight in males and decreased birth weight in females. In an experiment testing toxicity to SH-SY5Y neuroblastoma cells, oxybenzone and enzacamene were still prevalent in high concentrations 24 hours after the initial exposure. These ingredients increase lactate dehydrogenase release and reduce cellular metabolic activity, as designated through the MTT assay. This shows that oxybenzone and enzacamene can potentially harm neural cells as well as endocrine modulation [13].

In a trial with 24 healthy adults, sunscreens containing oxybenzone, avobenzone, octocrylene, and ecamsule were applied at a rate higher than the Food and Drug Administration's (FDA) recommended: 5 ng/ml of systemic absorption over seven days. Data indicated that none of these sunscreens inhibited CYP3A4 or CYP2D6, but enzacamene, oxybenzone, and trolamine inhibited CYP2C9. A concern is that CYP450 induction can affect many immunomodulating drugs [14].

Sunscreens are also able to be skin absorption enhancers, particularly for benzene derivatives. A study conducted with 2,4-dichlorophenoxyacetic acid, a popular herbicide that is classified by the International Agency for Research on Cancer as a carcinogen to humans, was applied to mouse skin in vitro. Octyl methoxycinnamate, oxybenzone, sulisobenzone, OD-PABA, octisalate, homosalate, and the insect-repellent DEET were tested. Out of the five sunscreen ingredients tested, only octocrylene did not enhance uptake [12]. This is concerning for farmers who may use the herbicide while simultaneously using sunscreens with any of these ingredients.

Chemicals that do not appear to cause endocrine system modulation include avobenzone, Tinosorb® M, Tinosorb® S, and octocrylene. Octocrylene does not show any adverse effects on the endocrine system. Octocrylene is mainly absorbed through the stratum corneum, is not teratogenic, and is considered a safe ingredient at or below a 10% concentration [11].

Sunscreen manufacturers use the FDA guideline ISO 24443:2021, which was derived from ISO 2443:2012, to determine the SPF measurements in their products. This entails preliminary sunscreen laboratory testing to approximate the SPF, but it is inaccurate when applied to the skin in the real world. ISO 24443:2021 allows for sunscreen testing to be conducted in vivo through testing subjects who are monitored for post-sun exposure erythema 24 hours after exposure [3].

Sunscreens can protect against UVA radiation and UVB radiation. Sunscreens labeled broad-spectrum are designed to protect against both UVA and UVB; however, SPF only measures erythema 24 hours post-exposure, which is produced by UVB radiation. Products that do not advertise a broad spectrum or protect against UVA and UVB may only protect against UVB radiation, leaving the consumer vulnerable to UVA radiation [2]. Oxidative damage from UVB is much lower than that from UVA radiation. Since SPFs generally measure protection against UVB, they do not accurately predict UVA protection [3,6].

A study comparing advertised sunscreen SPF and actual in vitro SPF was conducted to gauge the accuracy of ISO 24443:2012 testing with their respective SPFs. A total of 51 sunscreens were analyzed. SPF 15 was the standard evaluated alongside the test products. The results indicated that the mineral filter, ZnO, averaged a labeled SPF of 40 and only measured 17.6 in vitro. Another mineral filter with ZnO and TiO_2_ averaged a labeled SPF of 48.6 but an SPF of 44.6 in vitro. The mineral filter TiO_2_ averaged a labeled SPF of 35 but an SPF of 37.7 in vitro; mineral and organic sunscreens combined together were labeled SPFs of 40.2 but an SPF of 20.3 in vitro. Organic sunscreen had a labeled SPF of 58 but only an SPF of 29.8 in vitro [3].

With current FDA standards, 94% of the sunscreens tested would be approved, 67% would pass the UVA1/UV test, and only 35% would pass the European Union’s more strict requirements for labeling SPF. Products that contained TiO_2_, commonly ZnO and TiO_2_ combination sunscreen, were significantly better at UVA protection and holding true to their advertised SPF values while increasing SPF compared to ZnO alone [3].

In a randomized clinical trial, sunscreens had a limited effect on the development of squamous cell carcinoma and a clinically insignificant effect on the development of basal cell carcinoma. Since melanomas are primarily caused by repeated painful sunburns, sunscreens protect from melanoma when used correctly [7,8]. A Cochrane review showed no qualitative evidence that sunscreen reduces keratinocyte neoplasm. Limited and low-quality evidence indicates that sunscreens do not protect against the development of actinic keratosis or solar keratosis but may help with their progression in immunocompromised patients [7].

Other sunscreen ingredients are emerging with improving technology. Natural compounds like botanicals, non-botanicals, and biologics are added to sunscreens to increase their efficacy. The FDA considers these inactive ingredients because they do not directly impact the sunscreen's SPF [6]. Topical antioxidants are a powerful adjuvant to sunscreens to reduce non-melanoma cancers and ROS. Plus, they do not have to remain on the skin during sun exposure [5].

Lignin, sylmarin, and afzelin are naturally derived ingredients that increase the antioxidant properties of sunscreen while enhancing the SPF of the original sunscreen ingredients. They have powerful antioxidant capabilities to neutralize ROS from UV exposure. They are able to regulate the T-helper cell 1 (Th1) and T-helper cell 2 (Th2) immune responses to also lower inflammation [15,16].

Application and Risk Behaviors

The American Academy of Dermatology and the American Osteopathic College of Dermatology recommend that at least one ounce of sunscreen is needed to adequately cover a person’s body [17,18]. Application density impacts the final SPF, regardless of the advertised SPF, to a degree. Applying a higher amount of sunscreen than one ounce to an entire body shows it has higher protective benefits. Many people do not apply the 2 g/cm^2^ recommendation for sunscreens and may get a much lower SPF than advertised [6]. A study was done using three-dimensional photograph analysis. After applying 1.33 mg/cm^2^, the efficacy of SPF 15 was 23.8, SPF 30 was 47.5, and SPF 50 was 78.2 [19]. More studies are needed to accurately determine what levels of sunscreen application result in lower or higher SPF values.

Sunscreen holds up well when applied with other products simultaneously. In a study, participants were given SPF 16 and 50 to apply and moisturizer to apply, and a UV-emitting camera flash was used to test the absorption of the SPF. The preliminary study demonstrated that applying moisturizer did not decrease the SPF efficacy. There was no significant increase in sun protection with the amount of sunscreen applied, and there was no difference in protection if the sunscreen was applied before or after moisturizer was applied [20].

Sunscreen has the profoundly unique property of providing sun protection while underwater. However, the water expelled through the skin through sweat has a substantial deleterious effect on the sun's protective factor. To test this, hydrophobic compounds, which mimic sweat, were applied to a gelatin-based skin layer that replicated the thickness and texture of the skin. Laser-etched artificial pores into each side of the skin. Sunscreens were applied at 2 g/cm^2^, and cross-sectional images, 3D images, and z-resolution images to 10 µm were taken [21].

Results indicate that the bond of the sunscreen to the skin was a key factor in initial sunscreen protection. Once the skin was allowed to sweat, sunscreen was washed off, which resulted in a patchy droplet appearance on the skin. The sunscreen redistributes itself on the skin, leading to excessive amounts of sunscreen in some locations and minimal amounts of sunscreen in others. Sweat droplet expansion also shows evidence of physically pushing the sunscreen/skin barrier out and can even lead to a barrier burst [21]. With the redistribution, UV radiation penetrated the skin, where sunscreen was no longer present.

Studies have analyzed behavioral differences in sunscreen and sun protection use. Glenn et al. reported that only 46% of parents who were melanoma survivors discussed sun exposure and protection with their children’s physicians [22]. Another study was conducted with adolescents across Colorado, California, and Hawaii to determine their perceived risk of UV exposure. They varied in their perception of skin cancer, tanning, and the usage of sun protective measures. In the study, males were more likely to wear shirts with sleeves and hats but were less likely to perceive themselves to sunburn easily [22,23]. Females thought they sunburned easily but were more likely to stay in the shade, use sunscreen, and wear sunglasses [22,23]. As age increased, regardless of sex, the amount of sunscreen worn decreased. Participants with lighter skin or hair were more likely to wear sunscreen, hats, sunglasses, and protective clothing than participants with darker skin or hair. The usage of sunscreen was positively correlated with the perception of easy sunburn, but no other measurement of protection was significantly correlated. Tanning, wearing sunglasses, and the frequency of tanning increased with age; however, tanning was also the highest for participants with light skin [23].

Discussion

Protective barriers like sunscreen are beneficial in preventing UV radiation-induced melanoma and immunosuppression. However, with the large variety of sunscreen available today, no single sunscreen is best for everyone. A tailored approach to sunscreen ingredient recommendations is needed to ensure patients have access to safe UV protection.

Choosing a sunscreen with the highest efficacy depends on the ingredients. Inorganic sunscreens with titanium dioxide present the truest in vivo SPF as compared to the advertised SPF on the bottle. Overall, inorganic sunscreen is safe and well tolerated. By forming a physical barrier on the skin, inorganic sunscreen blocks UV radiation and heat, keeping the skin cooler outside.

Organic sunscreen ingredients should be chosen carefully. Some, like octocrylene, are safe and effective. Others, like oxybenzone, are dangerous to human health. Organic sunscreen's advantages are its ability to absorb into the skin and create a better skin feeling and physical look than inorganic sunscreen.

Adherence to a sunscreen regimen is just as important as choosing the right product. By underapplying inorganic sunscreen due to its white haze appearance on the skin, potential hesitancy from users exposes them to harmful UV radiation. Organic sunscreen offers cosmetic and UV radiation protection while leaving considerably less residue.

## Conclusions

Barrier sunscreens like titanium dioxide and zinc oxide are the most well-tolerated products with the fewest potential adverse effects. However, because they can leave behind a white, hazy residue, they are less preferred due to the look and feel on the skin. Absorbable sunscreens like avobenzone and octocrylene are very popular due to their lack of white haziness upon absorption into the skin, better feeling on the skin overall, and minimal adverse effects. There are other sunscreens, like oxybenzone, which should not be used due to adverse neuroendocrine modulation. Overall, finding a sunscreen that patients will use is patient-specific, and adherence and reapplication are two factors that are equally important as the product itself. More studies on sunscreen efficacy, adverse effects, and protection from UV-induced damage should be conducted to contribute to public health.
